# Supplementary material for: A simple pressure-assisted method for MicroED specimen preparation
Source: Nat Commun. 2021 Aug 19;12:5036. doi: 10.1038/s41467-021-25335-7 (PMC8377027; doi:10.1038/s41467-021-25335-7)
Supplement: Supplementary file 1 — Supplementary information [file 41467_2021_25335_MOESM1_ESM.pdf]

A simple pressure-assisted method for MicroED specimen preparation

Jingjing Zhao[1], Hongyi Xu[1*], Hugo Lebrette[2], Marta Carroni[2,3], Helena Taberman[4,5], Martin Högbom[2] & Xiaodong Zou[1*]

[1]Department of Materials and Environmental Chemistry, Stockholm University, Stockholm, Sweden

[2]Department of Biochemistry and Biophysics, Stockholm University, Stockholm, Sweden

[3]Science for Life Laboratory, Stockholm University, Solna, Sweden

[4]Max-Delbrück-Centrum for Molecular Medicine, Berlin, Germany

[5]Macromolecular Crystallography, Helmholtz-Zentrum Berlin für Materialien und Energie, Berlin, Germany

[*]Correspondence: hongyi.xu@mmk.su.se, xzou@mmk.su.se

Supplementary Methods

Preassis is widely applicable for MicroED specimen preparation, including crystals grown in low-viscous buffer conditions to highly-viscous PEG-rich crystallization conditions. It must be noted that different crystal samples grown in different conditions and with different sizes or shapes behave differently. The parameters for the method described here may need to be fine-tuned accordingly. Furthermore, the pressure range also depends on the size of the Büchner flask and the type of pump machine. Therefore, the parameters applied here for different types of protein crystal samples may need to be adjusted for a new setup. The specimen preparation comprises the following steps:

a. providing an EM grid;
b. applying a sample suspension onto the EM grid;
c. providing a pressure gradient through the EM grid in order to pull a portion of the liquid through the grid while removing the excess liquid with a filter paper on the other side of the grid;
d. plunging the specimen into a cryogenic bath.

Details of the setup and the specimen preparation procedure are described below.

Equipment

- Micropipette and tips, 0.5-10 μL (Eppendorf Research plus variable micropipette, #3116000015 or similar; Eppendorf tips 0.1-10 μL, # Z741098-960EA or similar)
- Tweezers (Dumont Tweezer, style5 #72705-01 or similar)
- Filter paper (Munktell Filtrak™ Grade3, 55 mm diameter, or similar)
- Quantifoil holey carbon grids (R 1.2/1.3, R 2.0/1, R 3.5/1, R2/2, or similar)
- Glow-discharge (PELCO easiGlow™ 9100 or similar)
- Cryo grid box (CGB4-1 SWISSCI Cryo grid box or similar)

- FEI coolant container
- Ethane and liquid nitrogen
- Vacuum aspirator or pump machine (PC 3001 VARIO)
- Polymer hose (hose, PVC-/latex-, 6 × 9 mm)
- Pressure meter (PELCO^{R} 2245 Minl Hot Vac. if a vacuum aspirator is used)
- Büchner flask (GLASSCO 500 mL, or similar)
- Home-made humidity chamber with a size of 55 cm × 40 cm × 50 cm. Digital
- thermos-hygrometer (KLIMA GUARD 30.5010, or similar)
- Humidifier (Sensky Aroma Diffuser (500 ml), or similar)

Procedure

1. Prepare liquid ethane using the FEI coolant container. Open the humidifier if a humidity control is desired. Since the front door of the humidity chamber is covered by two pieces of plastic films to provide an operation window and keep the humidity at the same time, the highest humidity that can reach is ~ 80%.

2. Glow discharge the grids (current 20 mA, glow 60 s, hold 10 s; can be changed if necessary). The hole size of the grid should be slightly smaller than the crystal size if possible. For very viscous samples, grids with large hole sizes are recommended, such as Quantifoil R 3.5/1 grids.

3. Put a filter paper on top of the flask.

4. Use a pair of tweezers to pick up a glow discharged grid and put it onto the center of the filter paper with the carbon side facing up.

5. Turn on the tap (if a vacuum aspirator is used) or start the vacuum pump. If a vacuum aspirator is used, a pressure meter needs to be installed to measure the pressure. For non-

viscous samples, typical pressures used for MicroED specimen preparation are around 20 mbar if a Quantifoil R1.2/1.3 is used (the pressure is not always necessary when a grid with larger hole size is used, e.g. Quantifoil R2/1 and R3.5/1). For viscous samples, a pressure of 70 mbar or higher may be needed even for grids with large hole sizes, e.g. Quantifoil R3.5/1.

6. Use a micropipette to take 3 μl of the sample, apply it onto the grid. The droplet will be dispersed through the grid immediately. Pick the grid up and plunge-freeze it within 5 to 10 s. For very viscous samples, one can decrease the sample volume to 1 or 2 μl, use a grid with large hole size (e.g. Quantifoil R 3.5/1), increase the pressure strength, or wait for a slightly longer time before picking up the grid. The manual plunging-freezing could affect the freezing speed and therefore the vitrification. If the original crystal suspension does not contain any cryo-protectants (e.g high molecular weight polymers) or has a relatively low salt concentration, it may be necessary to add a suitable cryo-protectant to minimize the chance for crystalline ice formation.

7. Cryotransfer the grid into a cryo-grid box.

In this setup, a vacuum aspirator with water-flow or a vacuum pump machine (recommended) can be used to produce the pressure/suction. The suction time may also affect the thickness of the vitrified ice. In this work, the suction time was kept at ~ 5 s for non-viscous crystal suspensions and ~10 s for viscous crystal suspensions. Based on the current setup of Preassis, the time is less controllable (due to manual grid handling) compared to the other two parameters pressure and hole size of the carbon film. A detailed study will be performed when a more controllable and automated setup is built. We admit that there is a small deviation between specimens prepared using the same parameters, which may result from the manual picking, time controlling, and also the contact between the grid and filter paper. A few trials are often

needed to find the suitable specimen preparation conditions for a new protein crystal sample, which takes a few hours including specimen preparation and grid screening on a TEM using a cryo-transfer holder. If we handle the process properly, the deviation is small and will not affect the overall results. In the future, new implementations, such as automation development and humidity chamber, may improve the throughput and reproducibility of the specimen preparation by Preassis. Furthermore, a vertical setup of Preassis (the grid is held vertically and a suction tube is placed behind the grid) can be implemented as an add-on to the Vitrobot to enable environmental control and automated plunge-freezing. Preassis can be also applied to pre-clipped EM grids used for auto-loading, which makes this method very promising for future automation.

Supplementary Figures

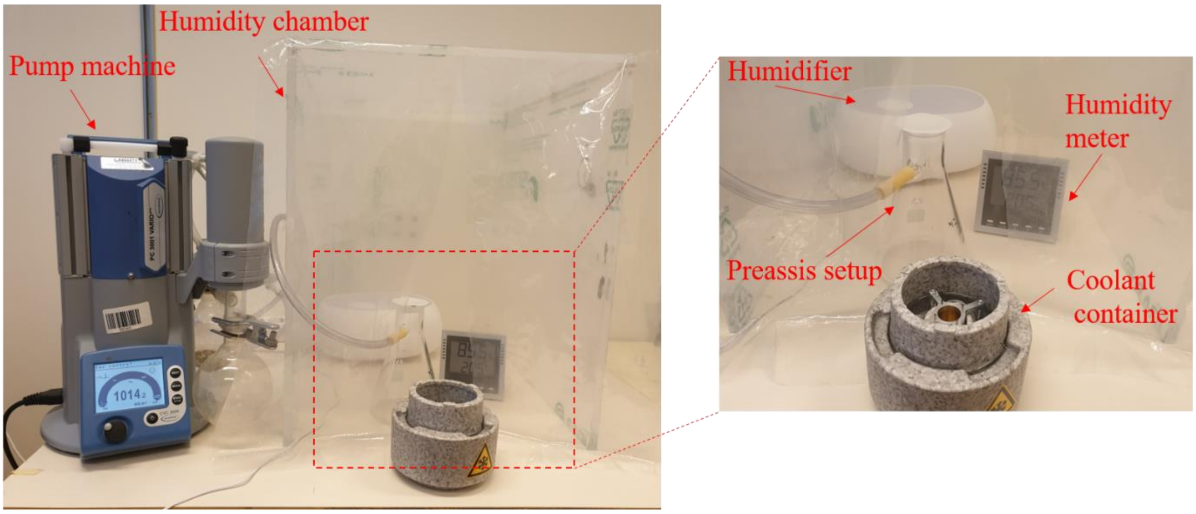

Supplementary Fig. 1

A simple setup of Preassis with a humidity chamber for MicroED specimen preparation, where a humidity up to ca 80% can be achieved. The humidity is controlled by a humidifier.

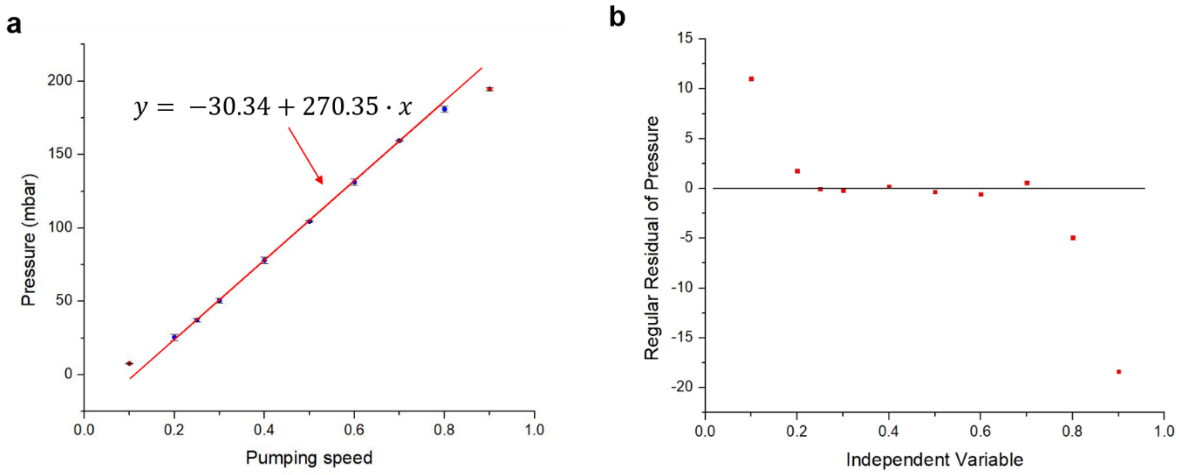

Supplementary Fig. 2

Relationship between the pumping speed and the resulting pressure of the pumping machine (PC 3001 VARIO).

a-b, The pressure is almost linearly related to the pumping speed in the region where the pumping speed is between 20% and 80%. The regular residual of the pressure in **b** is defined by the difference between the predicted value by $y = -30.34 + 270.35 \cdot x$ in **a** and the experimental value. The pressure was calculated from the difference between the measured atmospheric pressure (~ 1022 mbar) and the experimental pressure. At each pumping speed, the pressure was measured three times (sample size n = 3). Data are represented as mean values ± s.d.

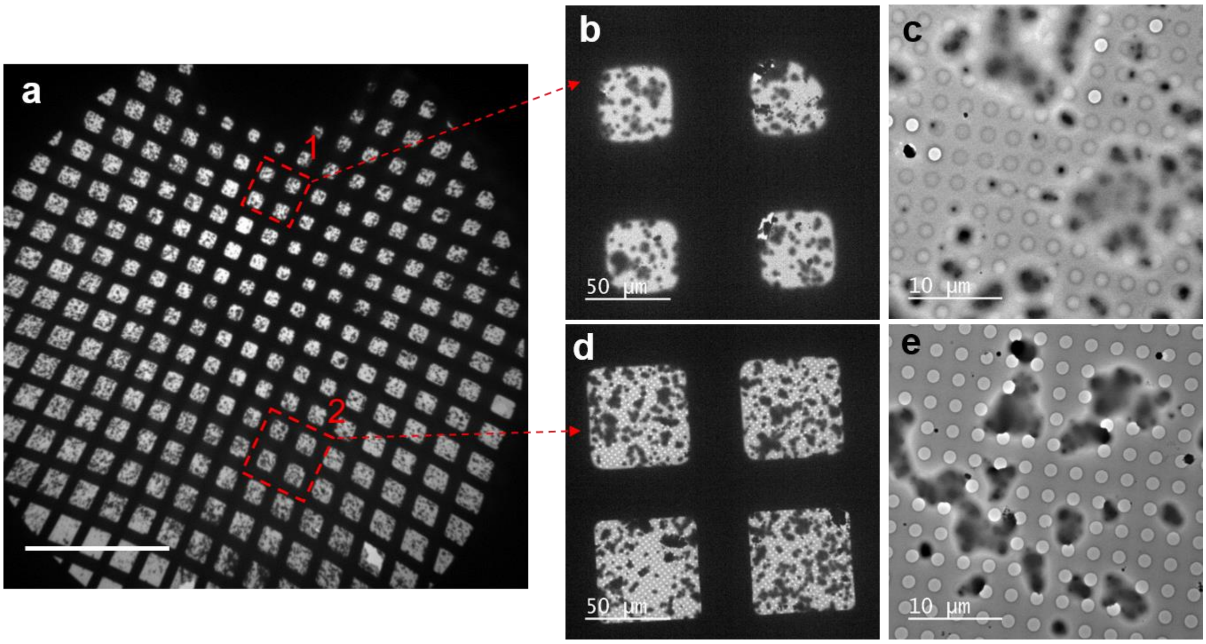

Supplementary Fig. 3

Ice thickness distribution across the grid prepared by Preassis from the tetragonal lysozyme crystal sample.

a, Low magnification image showing the inhomogeneous distribution of ice thickness on the entire TEM grid. The image is distorted especially at the edges because of the geometrical distortion of lenses at such a low magnification. The periodicity (86 µm) of grid squares is therefore used as a reference scale. The scale bar corresponds roughly to 500 µm. **b-c** and **d-e**, Two representative examples of areas with relatively thick (**b-c**) and thin (**d-e**) ice layers. This inhomogeneous distribution of ice thickness is a common feature of the specimens prepared by Preassis and it is confirmed by all the examples shown in the manuscript.

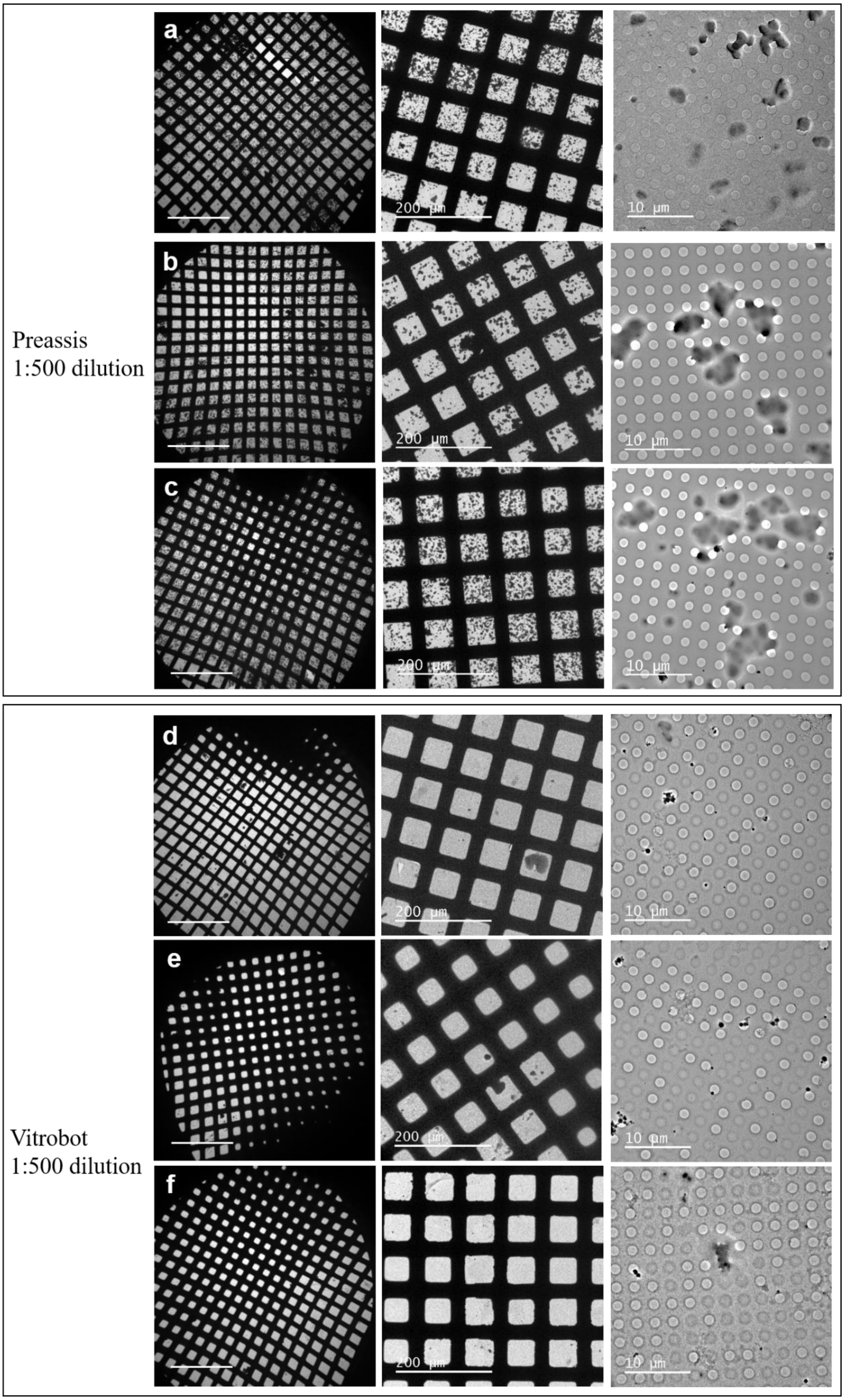

a
b
c
Preassis
1:500 dilution
d
e
f
Vitrobot
1:500 dilution
200 μm
10 μm

Supplementary Fig. 4

Comparison of crystal density between the grids prepared by Preassis and Vitrobot (Mark IV) using the same tetragonal lysozyme crystal sample.

a-c, TEM images of three MicroED specimens prepared by Preassis using the same experimental parameters: 3 µl droplet, Quantifoil grid R 1.2/1.3, 37.2 mbar pressure, suction time ca 5 s, room temperature (~ 20 °C), and humidity (~ 35%). **d-f**, TEM images of three MicroED specimens prepared by Vitrobot (Mark IV) using the same experimental parameters: 3 µl droplet, Quantifoil grid R 1.2/1.3, single blot, blotting force 5, blotting time of 5 s, room temperature (~ 20 °C) and humidity (~80%). All these grids were prepared from the same tetragonal lysozyme crystal suspension which was 500 × diluted from the original crystal suspension. These images were collected on a JEOL JEM-2100LaB_6 microscope equipped with a Gatan Orius camera. The images in the left column are distorted especially at the edges because of the geometrical distortion of lenses at such a low magnification. The periodicity (86 µm) of grid squares is therefore used as a reference scale. The scale bar corresponds roughly to 500 µm.

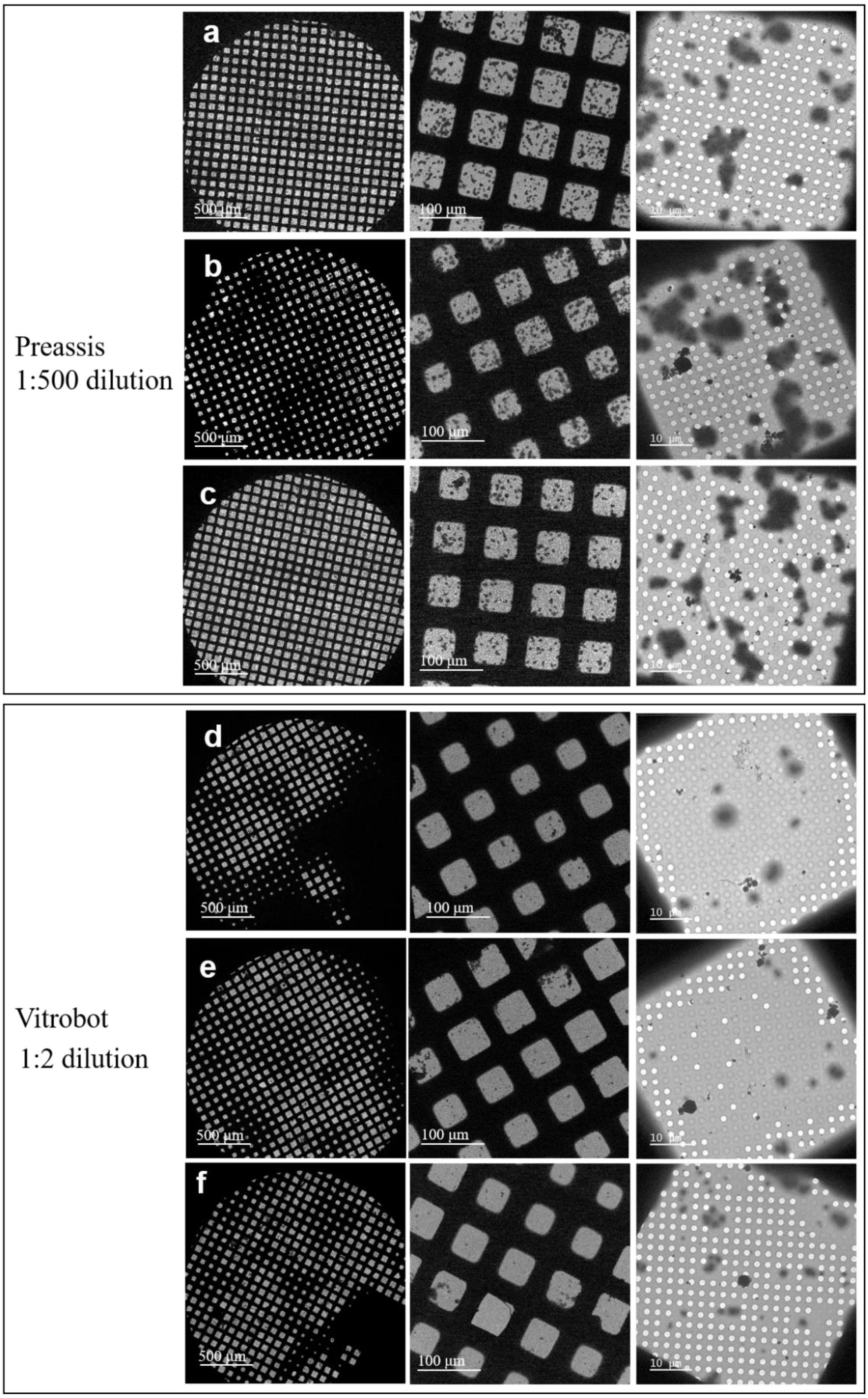

Supplementary Fig. 5

Comparison of crystal density between the grids prepared by Preassis and Vitrobot (Mark IV) using 500 times and 2 times diluted tetragonal lysozyme crystal suspensions, respectively.

a-c, TEM images of three MicroED specimens prepared by Preassis using the same experimental parameters: 3 µl droplet, Quantifoil grid R 1.2/1.3, 37.2 mbar pressure, suction time ca 5 s, and room temperature (ca 20 °C) and humidity (80%). A 500 × diluted crystal suspension was used. **d-f**, TEM images of three MicroED specimens prepared by Vitrobot (Mark IV) using the same experimental parameters: 3 µl droplet, Quantifoil grid R 1.2/1.3, single blot, blotting force 5, blotting time of 5 s, room temperature (~ 20 °C) and humidity (~ 80%). A 2 × diluted crystal suspension was used. These images were collected on a Themis Z microscope (300 kV) equipped with a Gatan OneView camera.

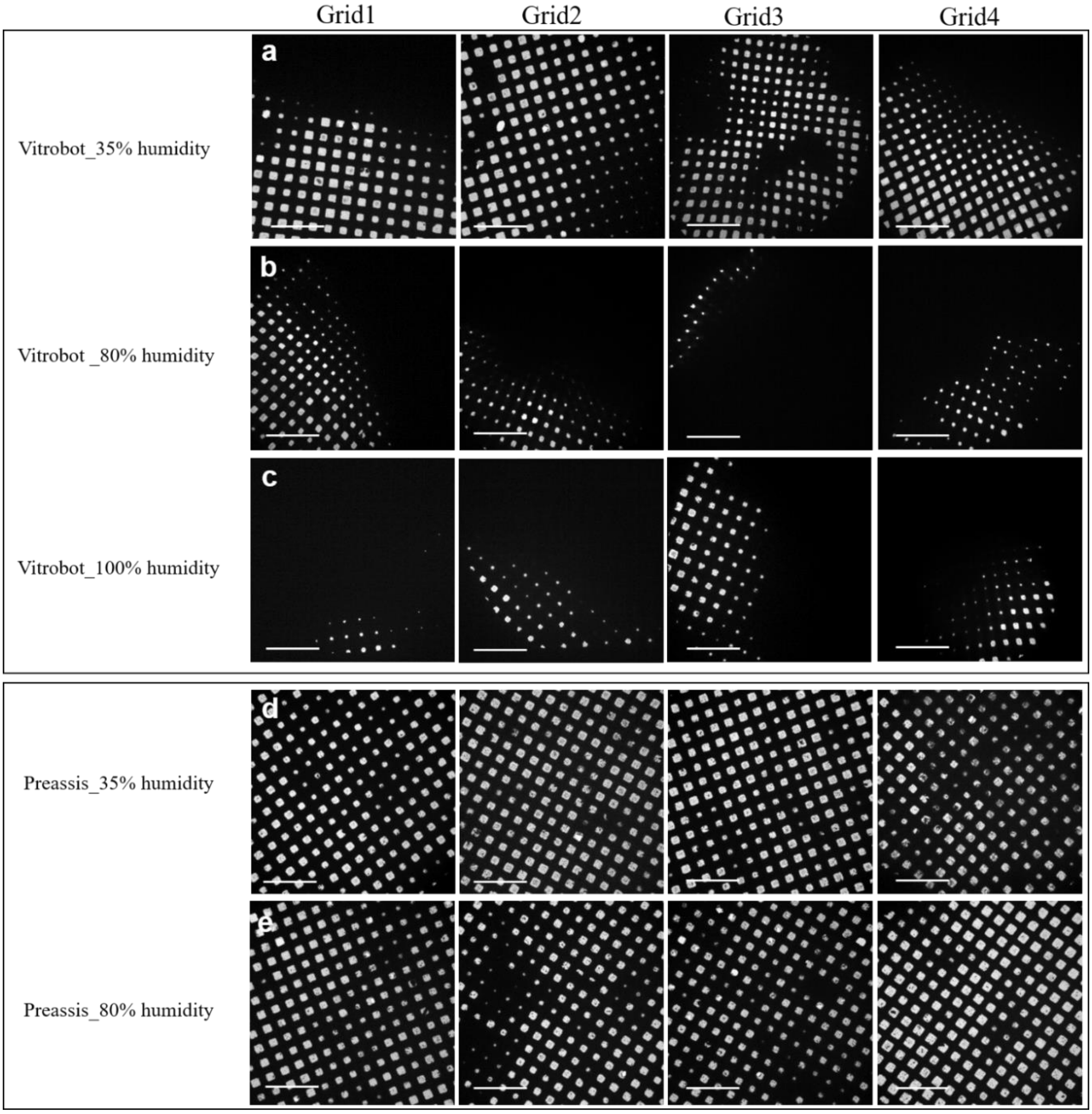

Supplementary Fig. 6

Comparison of EM grids prepared by Preassis and Vitrobot under different humidity using a mixture of ZSM-5 microcrystals and 40% PEG 400.

a-**c**, Low magnification images of MicroED specimens prepared by Vitrobot (Mark IV) with blotting force 5 (5 s) and humidity of 35%, 80%, and 100%, respectively. **d** and **e**, Low magnification images of MicroED specimens prepared by Preassis with a pressure of 180 mbar (5 s) and humidity of 35% and 80%, respectively. The EM grids (Quantifoil R 3.5/1) were prepared at room temperature (~ 20 °C). Each experiment condition was repeated by ≥ 4 times. Due to the current design of the humidity control box for the Preassis setup (Supplementary Fig. 1), the humidity can only reach ~ 80%. The images were collected on a JEOL JEM-2100LaB$_6$ microscope equipped with a Gatan Orius camera. The images are distorted especially at the edges because of the geometrical distortion of lenses at such a low

magnification. The periodicity (86 µm) of grid squares is therefore used as a reference scale. The scale bar corresponds roughly to 500 µm.

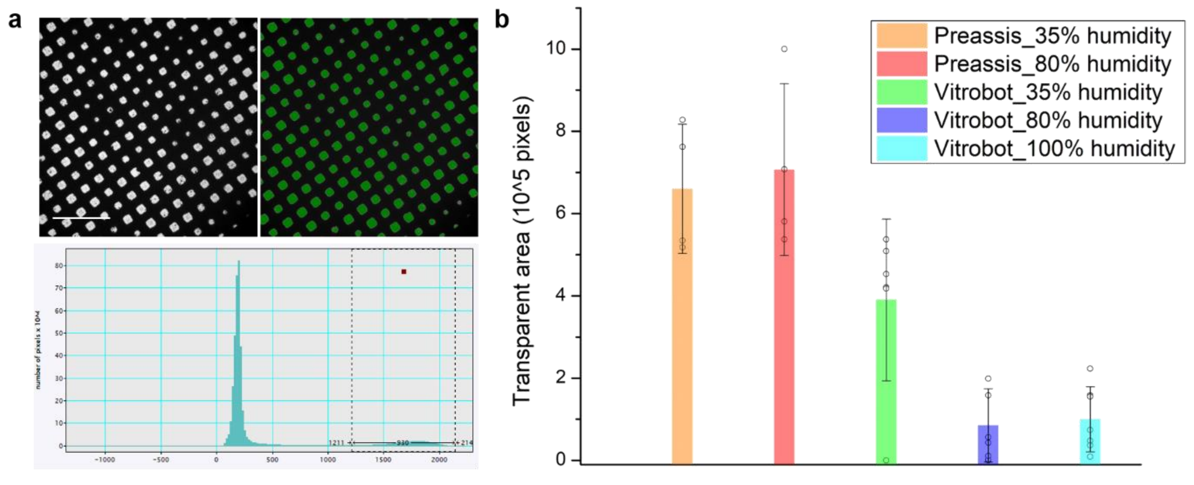

Supplementary Fig. 7

Quantitative analysis of the vitrified ice thickness of specimens prepared by Preassis and Vitrobot under different humidity using a mixture of ZSM-5 microcrystals and 40% PEG 400.

This analysis is based on the specimens described in Supplementary Fig. 6. The transparent area of each grid (presented in the number of pixels), as marked in green in **a**, was extracted using DigitalMicrograph. The sum of the transparent area of each grid is used to compare the overall ice thickness of the grid. The transparent areas of grids prepared under different specimen preparation conditions are shown in **b**. For grids prepared by Preassis, nearly all grid squares are transparent, and the humidity does not make a significant influence. For grids prepared by Vitrobot, the average transparent areas have been significantly reduced by ~ 40% under 35% humidity and ~ 88% under 80% humidity. The effect of the humidity on the ice thickness is obvious when using Vitrobot. By Preassis, each specimen preparation condition was repeated four times (sample size n = 4); by Vitrobot, specimen preparations at 35%, 80%, and 100% were repeated six times (n = 6), eight times (n = 8), and seven times (n = 7), respectively. Data are represented as mean values ± s.d. The scale bar corresponds roughly to 500 µm.

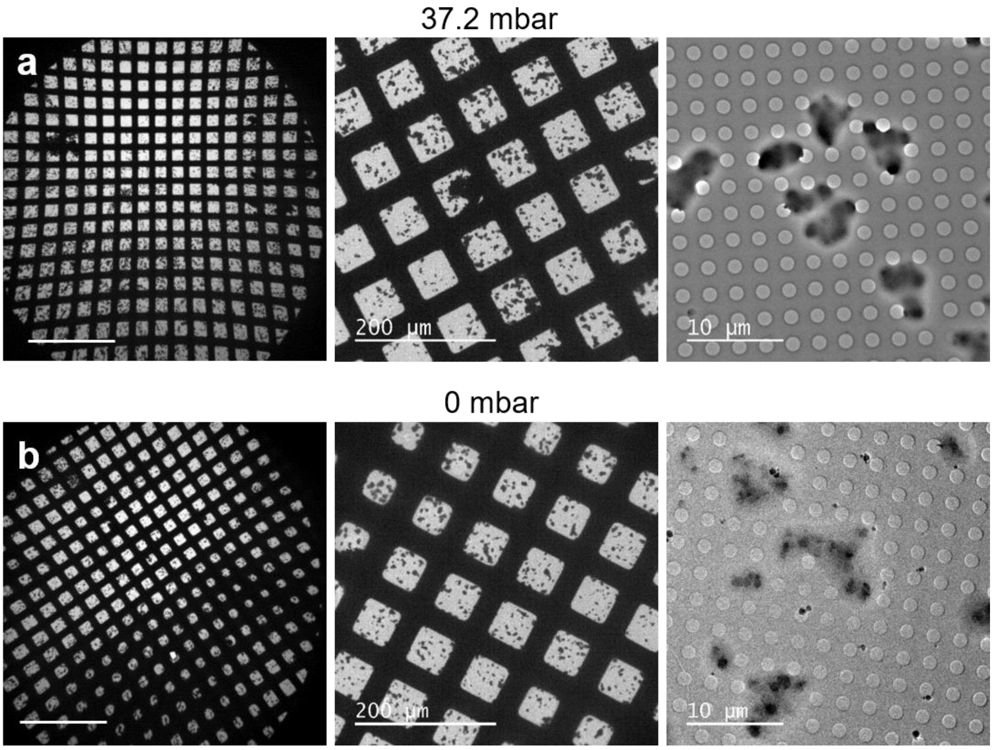

Supplementary Figure 8

Influence of pressure on MicroED specimen prepared from a non-viscous buffer by Preassis using a tetragonal lysozyme crystal sample.

a and **b**, Low magnification images and high magnification images taken from the specimens prepared with pressure of 37.2 mbar and 0 mbar, respectively. For these specimens, tetragonal lysozyme crystal sample and Quantifoil grid R 1.2/1.3 were used. The images on the left are distorted especially at the edges because of the geometrical distortion of lenses at such a low magnification. The periodicity (86 μm) of grid squares is therefore used as a reference scale. The scale bar corresponds roughly to 500 μm. Each specimen preparation condition was repeated more than three times with similar results.

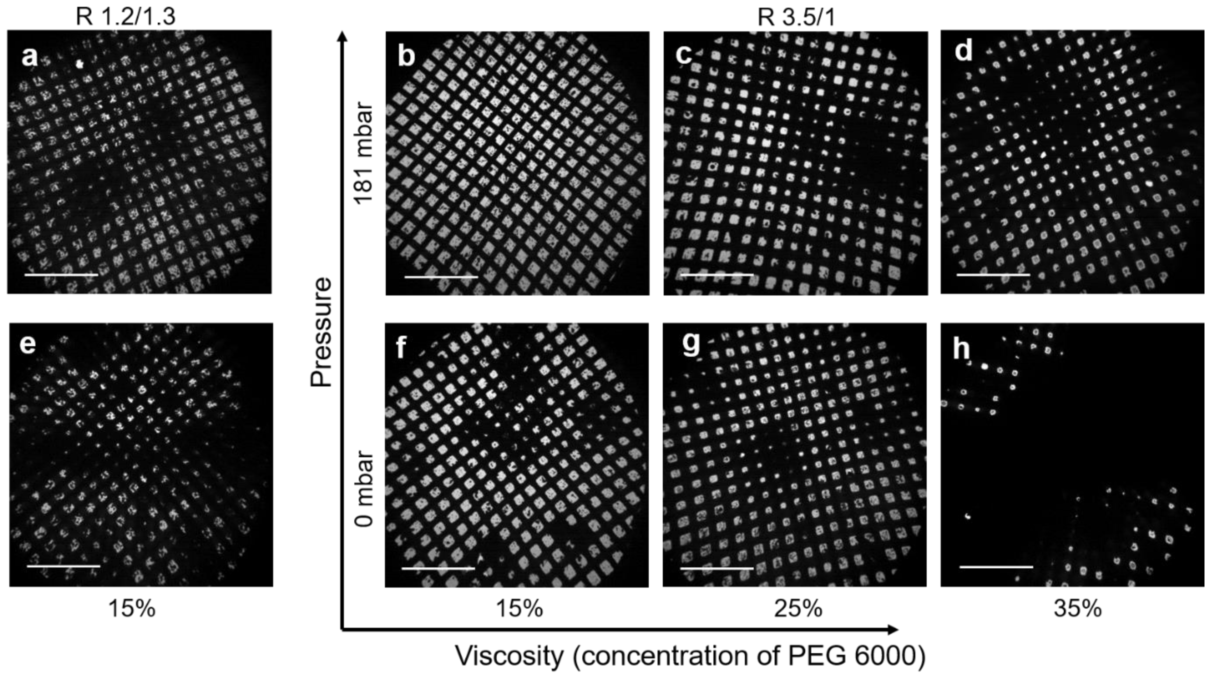

Supplementary Figure 9

Influence of pressure and hole size on MicroED specimens prepared by Preassis using crystal suspensions with different viscosity.

a and **e**, Low magnification TEM images taken from the specimens prepared from a crystal suspension with 15% PEG 6000 using Quantifoil grids R 1.2/1.3 with 181 and 0 mbar pressure, respectively. **b**-**d** and **f**-**h**, Low magnification TEM images taken form the specimens prepared by Preassis from crystal suspensions with different viscosity (15%, 25%, and 35% PEG 6000) using Quantifoil grids R 3.5/1 at 181 and 0 mbar pressure, respectively. The crystal suspensions with different viscosities were made by mixing ZSM-5 microcrystals with different concentrations of PEG 6000. The images were collected on a JEOL JEM-2100LaB_6 microscope equipped with an Orius detector. The images are distorted especially at the edges because of the geometrical distortion of lenses at such a low magnification. The periodicity (86 μm) of grid squares is therefore used as a reference scale. The scale bar corresponds roughly to 500 μm. Each specimen preparation condition was repeated three to five times with similar results.

Supplementary Table1

Statistics of the MicroED data of tetragonal lysozyme crystals collected from cryo-EM grids prepared by Vitrobot (Mark IV)

	Data	Resolution (Å) [a]	I/Sigma [a]	R_{meas} [a]	$CC_{1/2}$ [a]	*a* (Å) [b]	*b* (Å) [b]	c (Å) [b]
Grid1	1	35.17-2.73 (2.83-2.73)	5.6(1.0)	0.344(1.675)	0.973(0.241)	78.63(5)	78.63(5)	39.17(58)
	2	35.17-2.68 (2.78-2.68)	5.4(1.0)	0.293(1.463)	0.969(0.236)	78.63(2)	78.63(2)	39.36(47)
	3	35.19-2.80 (2.90-2.80)	4.0(1.0)	0.360(1.447)	0.950(0.242)	78.69(13)	78.69(13)	39.31(83)
Grid2	4	35.15-2.61 (2.70-2.61)	4.9(1.0)	0.313(1.667)	0.963(0.166)	78.58(6)	78.58(6)	38.77(29)
	5	35.18-2.72 (2.82-2.72)	5.1(1.0)	0.340(1.635)	0.965(0.295)	78.67(2)	78.67(2)	38.68(51)
	6	35.24-2.60 (2.69-2.60)	5.6(1.0)	0.268(1.541)	0.973(0.324)	78.79(5)	78.79(5)	38.97(20)
Grid3	7	35.14-2.50 (2.59-2.50)	5.0(1.0)	0.299(1.214)	0.972(0.544)	78.57(5)	78.57(5)	39.92(76)
	8	35.17-2.81 (2.91-2.81)	4.5(1.0)	0.309(1.447)	0.966(0.447)	78.65(5)	78.65(5)	38.80(9)
	9	35.12-2.53 (2.62-2.53)	4.9(1.0)	0.302(1.380)	0.966(0.427)	78.53(7)	78.53(7)	39.88(85)
Average		2.67(11)[c]	5.0(5)[d]	0.314(29) [d]	0.966(7) [d]	78.64(8)	78.64(8)	39.21(46)

[a]Highest resolution shell is shown in parenthesis. The corresponding I/sigma, R_{meas}, and $CC_{1/2}$ at the highest resolution shell are shown in parenthesis.

[b]Standard deviations of the unit cell parameters are shown in parenthesis.

[c]Average highest resolution. Standard deviations are shown in parenthesis.

[d] Average I/sigma, R_{meas}, and $CC_{1/2}$. Standard deviations are shown in parenthesis.

Notes: The data resolution was cut to I/sigma ≥ 1. These grids were prepared by Vitrobot under 80% humidity. The grid images and specimen preparation conditions were shown in Supplementary Fig. 5d-f.

Supplementary Table2

Statistics of the MicroED data of tetragonal lysozyme crystals collected from cryo-EM grids prepared by Preassis

	Data	Resolution (Å) [a]	I/Sigma [a]	R_{meas} [a]	$CC_{1/2}$ [a]	*a* (Å) [b]	*b* (Å) [b]	c (Å) [b]
Grid1	1	35.29-2.50 (2.59-2.50)	6.0(1.0)	0.301(1.413)	0.975(0.520)	78.90(12)	78.90(12)	39.45(59)
	2	35.28-2.59 (2.68-2.59)	5.4(1.0)	0.300(1.220)	0.970(0.390)	78.88(4)	78.88(4)	38.80(48)
	3	35.28-2.45 (2.54-2.45)	5.7(1.0)	0.320(1.595)	0.975(0.120)	78.88(6)	78.88(6)	39.55(41)
Grid2	4	35.26-2.68 (2.78-2.68)	4.3(1.0)	0.316(1.348)	0.955(0.411)	78.83(4)	78.83(4)	39.58(12)
	5	35.25-2.70 (2.80-2.70)	4.4(1.0)	0.303(1.425)	0.966(0.402)	78.811(2)	78.811(2)	39.34(23)
	6	35.26-2.54 (2.63-2.54)	5.8(1.0)	0.310(1.423)	0.975(0.243)	78.84(9)	78.84(9)	39.21(58)
Grid3	7	35.31-2.58 (2.67-2.58)	4.8(1.0)	0.312(1.356)	0.93(0.463)	78.95(3)	78.95(3)	39.47(50)
	8	35.33-2.56 (2.65-2.56)	4.5(1.0)	0.334(1.454)	0.958(0.377)	79.00(3)	79.00(3)	38.21(26)
	9	35.27-2.55 (2.64-2.55)	4.8(1.0)	0.388(1.640)	0.966(0.260)	78.86(6)	78.86(6)	38.43(17)
Average		2.57(8) [c]	5.1(7) [d]	0.320(28) [d]	0.963(14) [d]	78.88(6)	78.88(6)	39.12(51)

[a]Highest resolution shell is shown in parenthesis. The corresponding I/sigma, R_{meas}, and $CC_{1/2}$ at the highest resolution shell are shown in parenthesis. The data resolution was cut to I/sigma ≥ 1

[b]Standard deviations of the unit cell parameters are shown in parenthesis.

[c]Average highest resolution. Standard deviations are shown in parenthesis.

[d] Average I/sigma, R_{meas}, and $CC_{1/2}$. Standard deviations are shown in parenthesis.

Notes: The data resolution was cut to I/sigma ≥ 1. These grids were prepared by Preassis under 80% humidity. The grid images and specimen preparation conditions were shown in Supplementary Fig. 5a-c.
