# Supplementary material for: A simple pressure-assisted method for MicroED specimen preparation
Source: Nat Commun. 2021 Aug 19;12:5036. doi: 10.1038/s41467-021-25335-7 (PMC8377027; doi:10.1038/s41467-021-25335-7)
Supplement: Supplementary file 3 — Description of Additional Supplementary Files [file 41467_2021_25335_MOESM3_ESM.pdf]

Description of Additional Supplementary Files

Supplementary Movie 1: The video was generated from a MicroED data of R2lox crystal. The specimen used for this data collection was prepared by Preassis with 30.7 mbar pressure and a Quantifoil grid R 3.5/1. The data was collected on a JEOL JEM-2100 (200 kV) microscope using selected-area electron diffraction mode. The data collection was done by continuously rotating the crystal with a rotation speed of 0.45 $°s^{-1}$ and exposure time of 2 s, and ED frames were simultaneously recorded by a fast Timepix hybrid pixel detector in movie mode.
