# Supplementary material for: A simple pressure-assisted method for MicroED specimen preparation
Source: Nat Commun. 2021 Aug 19;12:5036. doi: 10.1038/s41467-021-25335-7 (PMC8377027; doi:10.1038/s41467-021-25335-7)
Supplement: Supplementary file 5 — Reporting summary [file 41467_2021_25335_MOESM5_ESM.pdf]

nature research

Corresponding author(s): Hongyi Xu, Xiaodong Zou

Last updated by author(s): 27/06/21

Reporting Summary

Nature Research wishes to improve the reproducibility of the work that we publish. This form provides structure for consistency and transparency in reporting. For further information on Nature Research policies, see our Editorial Policies and the Editorial Policy Checklist.

Statistics

For all statistical analyses, confirm that the following items are present in the figure legend, table legend, main text, or Methods section.

n/a	Confirmed	
☐	☒	The exact sample size (n) for each experimental group/condition, given as a discrete number and unit of measurement
☐	☒	A statement on whether measurements were taken from distinct samples or whether the same sample was measured repeatedly
☒	☐	The statistical test(s) used AND whether they are one- or two-sided<br>*Only common tests should be described solely by name; describe more complex techniques in the Methods section.*
☒	☐	A description of all covariates tested
☒	☐	A description of any assumptions or corrections, such as tests of normality and adjustment for multiple comparisons
☐	☒	A full description of the statistical parameters including central tendency (e.g. means) or other basic estimates (e.g. regression coefficient) AND variation (e.g. standard deviation) or associated estimates of uncertainty (e.g. confidence intervals)
☒	☐	For null hypothesis testing, the test statistic (e.g. F, t, r) with confidence intervals, effect sizes, degrees of freedom and P value noted<br>*Give P values as exact values whenever suitable.*
☒	☐	For Bayesian analysis, information on the choice of priors and Markov chain Monte Carlo settings
☒	☐	For hierarchical and complex designs, identification of the appropriate level for tests and full reporting of outcomes
☒	☐	Estimates of effect sizes (e.g. Cohen's d, Pearson's r), indicating how they were calculated

Our web collection on statistics for biologists contains articles on many of the points above.

Software and code

Policy information about availability of computer code

Data collection	MicroED data were collected by Instamatic (version 1.0.0) and InsteaDMatic (version 1.0.0).
Data analysis	TEM images were analyzed by DigitalMicrograph (version 3.42.3050.0). MicroED data were processed and analyzed by XDS (version Feb 5, 2021 BUILT = 20210323).

For manuscripts utilizing custom algorithms or software that are central to the research but not yet described in published literature, software must be made available to editors and reviewers. We strongly encourage code deposition in a community repository (e.g. GitHub). See the Nature Research guidelines for submitting code & software for further information.

Data

Policy information about availability of data

All manuscripts must include a data availability statement. This statement should provide the following information, where applicable:
- Accession codes, unique identifiers, or web links for publicly available datasets
- A list of figures that have associated raw data
- A description of any restrictions on data availability

Source data for the plots shown in Supplementary Fig. 2 and Supplementary Fig. 7 are provided with this manuscript. A raw MicroED data of R2lox, collected from the specimen prepared by Preassis, is attached as a Supplementary video. Raw tetragonal lysozyme MicroED data (Supplementary Table1 and Table2) are available from the SBGrid Data Bank (doi: 10.15785/SBGRID/842). Other data are available from the corresponding authors upon reasonable request.

Field-specific reporting

Please select the one below that is the best fit for your research. If you are not sure, read the appropriate sections before making your selection.

☒ Life sciences ☐ Behavioural & social sciences ☐ Ecological, evolutionary & environmental sciences

For a reference copy of the document with all sections, see nature.com/documents/nr-reporting-summary-flat.pdf

Life sciences study design

All studies must disclose on these points even when the disclosure is negative.

Sample size	No sample-size calculations have been performed in this paper. Four crystal samples were chosen in order to show the applicability of Preassis which 1) can handle non-viscous crystal suspensions and obtain higher crystal density on an EM grid compared to Vitrobot (needle-shaped orthorhombic lysozyme and tetragonal lysozyme crystal suspensions); 2) can handle viscous crystal suspensions (R2lox and GTPase crystal suspensions).
Data exclusions	No TEM images of grids prepared by different methods under different conditions were excluded. Diffraction spots with intensities below a pre-defined threshold are automatically excluded by the integration software XDS since they are believed to contain only spurious information. The same signal/noise threshold (I/sigma ≥ 1) has been used for defining the highest data resolution in order to compare the data quality obtained from different specimen preparation methods.
Replication	Four different crystal samples were used to show reproducibility of Preassis with regards to handling protein crystals grown in various buffer conditions and samples with low crystal concentrations. Multiple grids (3-5) were repeated at each specimen preparation condition. MicroED data quality comparison was made based on several datasets (up to 10) collected from three grids prepared by each specimen preparation method. All attempts to replicate these specimen preparations and data collection and processing were successful.
Randomization	This is not related to our study. Our work is relate to a new MicroED specimen preparation method and it is no need to take the randomization of samples into account.
Blinding	Blinding is not relevant to our study. This work is related to a new method which doesn't introduce bias and no group allocation needs to be taken into account. Data analysis was exclusively software-based.

Reporting for specific materials, systems and methods

We require information from authors about some types of materials, experimental systems and methods used in many studies. Here, indicate whether each material, system or method listed is relevant to your study. If you are not sure if a list item applies to your research, read the appropriate section before selecting a response.

Materials & experimental systems

n/a	Involved in the study
☒	☐ Antibodies
☒	☐ Eukaryotic cell lines
☒	☐ Palaeontology and archaeology
☒	☐ Animals and other organisms
☒	☐ Human research participants
☒	☐ Clinical data
☒	☐ Dual use research of concern

Methods

n/a	Involved in the study
☒	☐ ChIP-seq
☒	☐ Flow cytometry
☒	☐ MRI-based neuroimaging
